# A Simple Incidence-Based Method to Avoid Misinterpretation of Bovine Tuberculosis Incidence Trends in Great Britain

**DOI:** 10.1371/currents.outbreaks.6fd216fc24317f2ce04a7c5705a30c69

**Published:** 2014-01-24

**Authors:** Isobel M. Blake, Christl A. Donnelly

**Affiliations:** Department of Infectious Disease Epidemiology, Imperial College London, London, UK; Department of Infectious Disease Epidemiology, Imperial College London, London, UK

**Keywords:** Bovine TB, Targeted surveillance

## Abstract

The incidence of bovine tuberculosis (TB) in Great Britain has generally been increasing in recent decades. Routine ante-mortem testing of cattle herds is required for disease surveillance and control, due to the asymptomatic nature of the infection. The Department for Environment, Food and Rural Affairs (Defra) publishes TB incidence trends as the percentage of officially TB-free (OTF) herds tested per month with OTF status withdrawn due to post-mortem evidence of infection. This method can result in artefactual fluctuations. We have previously demonstrated an alternative method, that distributes incidents equally over the period of risk, provides a more accurate representation of underlying risk. However, this method is complex and it may not be sufficiently straightforward for use in the national statistics. Here we present a simple incidence-based method that adjusts for the time between tests and show it can provide a reasonable representation of the underlying risk without artefactual fluctuations.

## Introduction

Bovine tuberculosis (TB) in Great Britain (GB) has generally been increasing in the last thirty years^1^ and has resulted in a huge strain on the cattle industry. In 2012, 37,735 cattle were compulsorily slaughtered due to detected herd TB incidents,^2^ resulting in compensation costs of approximately £30 million to farmers in England from the Department for Environment, Food and Rural Affairs (Defra) that year.^3^

Cattle infected with *Mycobacterium bovis* (the etiological agent of bovine TB) do not normally display clinical signs unless the infection is well advanced. Routine and targeted ante-mortem screening of herds using the tuberculin skin test is performed in GB for control and surveillance of bovine TB whereby officially TB-free (OTF) cattle herds are tested for evidence of exposure of *M. bovis* annually or every four years depending on past incidence of TB in a region (until December 2012 two-yearly and three-yearly testing frequencies had also been used)^4^ . In England, nearly 60% of herds are annually tested for TB (those in the West and Midlands), whereas the remainder are tested every four years, thus reflecting the regional clustered nature of the disease. All cattle herds in Wales have been tested annually since 2010, whereas Scottish herds are routinely tested every four years or are not tested at all (Scotland was declared officially free from bovine TB by the European Commission in September 2009). Unbiased interpretation of the data coming from this surveillance programme is crucial to understand the underlying incidence trends and thereby to inform discussion on the impact of the various control tools, including badger culling^5^ and vaccination.^6^

Defra currently publish the national statistics of GB bovine TB incidence as the monthly percentage of OTF herds tested that result in the OTF status being withdrawn (OTFW) since January 1996^2^ (Fig. 1a, red line). We have previously shown that this method can lead to artefactual fluctuations in the reported trends^7^ when large numbers of herds are moved to more frequent testing, and can therefore be misleading, if the downward part of these fluctuations are interpreted as a decline in the underlying incidence. Recent trends in the national statistics of the GB bovine TB incidence have been discussed in the current debate on the need for badger culling. To inform the debate most usefully, the incidence trends should be quantified using a method which is not liable to artefactual fluctuations.

We have previously presented an alternative method,^7^ adapted from a method presented by Cox,^8^ that distributes detected cattle herd incidents across the period between herd tests and provides a more accurate description or the underlying risk over time. However the method is somewhat mathematically complex to compute and it may not be sufficiently straightforward for use in the national statistics and for understanding by a lay audience.

At the beginning of 2014 it is expected that a public consultation will take place to decide how the national incidence trends of bovine TB in GB should be reported from the surveillance data. Here we present and apply a new simple incidence-based method that adjusts the monthly percentage of OTF herds resulting in OTFW breakdowns as a function of the time since the last herd test, which may effectively quantify the underlying incidence trends for the general public. We use simulations from a mathematical stochastic model of herd testing and infection incidence over time^7^ (re-fitted to include more recent data from 2010-2012 as well) to compare the current Defra reporting method, the previously published adapted Cox method^7^ and the simple incidence-based method in their ability to represent the model-estimated underlying trends.

## Methods

Data on the total number of OTF herds tested per month and the number of these tests that resulted in OTFW status from January 2003 to December 2012 for England, Wales and Scotland were obtained from the publicly reported national statistics published by Defra^2^ (Data given in Appendix 2).The numbers of tests that resulted in OTFW during May 2011 – December 2012 are still subject to final confirmation and are presented as a range for each month in the national statistics and so for this period the mid-point of the reported range was used. Note the data is not stratified by testing interval.

The stochastic dynamic model of herd testing and incidence of bovine TB in GB was described previously^7^ and was fitted to the national statistics data. The model contains a monthly risk of developing detectable *M. bovis* infection per herd. The times that the national incidence risk changed and the magnitude of changes were estimated (assuming a linear spline model for the underlying trend), along with the times that large-scale changes in testing frequencies occurred. The underlying model monthly risk is presented as the mean risk across all herds as a percentage, multiplied by twelve (Fig. 1b) to allow for direct comparison with the current Defra method. Model parameters were estimated using maximum likelihood estimation for the period January 2003 – December 2012. The likelihood of observing the data (number of OTFW breakdowns for a given month) was described previously^7^ , conditional on the model parameters and the number of observed OTF herds tested. One hundred stochastic simulations were run under the best fit model with a given underlying incidence risk per month (Fig. 1b), recording the number of OTF herds tested per month and the number of these that resulted in OTFW (i.e. representing 100 simulated national surveillance data-sets with a known underlying incidence risk per month).

Three methods were applied to these 100 stochastic simulations of herd testing to evaluate their ability to represent underlying incidence risk: the current Defra method (Fig. 1a, grey lines); the adapted Cox method (Fig. 1c); and the new simple incidence-based method which adjusts the current Defra method for the time spent between routine herd tests (Fig. 1d).

Defra publish the incidence trends over time as the percentage of OTF herds tested that result in an OTFW breakdown per month, smoothed using a 23-term Henderson moving average for seasonally-adjusted data (using the X-11 method from the freely available software http://www.census.gov/srd/www/x12a/ with the windows interface, version 3.0).

The adapted Cox method^7^ distributes incident events over the period of risk and allows for multiple introductions of infection during this period. The method can either assume all herds in each testing frequency are at equal risk to becoming infected or that there is heterogeneity in risk such that a given proportion of herds in each testing frequency are at risk of becoming infected. We previously found that decreasing the proportion of herds assumed to be at risk of becoming infected resulted in increased estimates of underlying risk but provided a similar temporal trend to assuming no heterogeneity in risk^7^ . Comparing estimates from assuming no heterogeneity in risk to assuming only1/5 of herds were at risk, provided sufficient bounds of the true underlying risk.

The third method we propose here is incidence-based in that it adjusts the current Defra method for the time period between tests. The incidence-based estimated risk per month (*t*), \begin{equation*}\hat{r}_{t}\end{equation*}, is given as: \begin{equation*}\hat{r}_{t}  = \frac{1}{\sum_{\tau =1}^{\tau =4}{N_{\tau ,t} } } \sum_{\tau =1}^{\tau =4}{\left( \frac{x_{\tau ,t} }{\tau } \right)  } \end{equation*}where *N*_*τ,t *_corresponds to the number of OTF herds tested in month *t *on testing frequency *τ*, and *x*_*τ,t *_denotes the number of these tests that resulted in an OTFW breakdown that were of testing frequency*τ*, (where*τ*= 1,2,3 or 4 (the number of years between herd tests)). Therefore for a given month annually tested herds contribute more to the month’s incidence compared to less frequently tested herds. The incidence trends were then smoothed using the 23-term Henderson moving average for seasonally-adjusted data and multiplied by 100 to give a percentage.

We did not apply the adapted Cox method or the new incidence-based method to the published GB data as these methods require the number of tests and positive tests to be stratified by testing interval, and this information is not publicly reported in the national statistics.

## Results

The smoothed monthly percentage of OTF herds tested that result in the OTF status being withdrawn, as presented by Defra as the national incidence trend, is shown in Fig 1a (red line).

**Comparison of different methods to estimate the underlying incidence risk of bovine tuberculosis in Great Britain.**
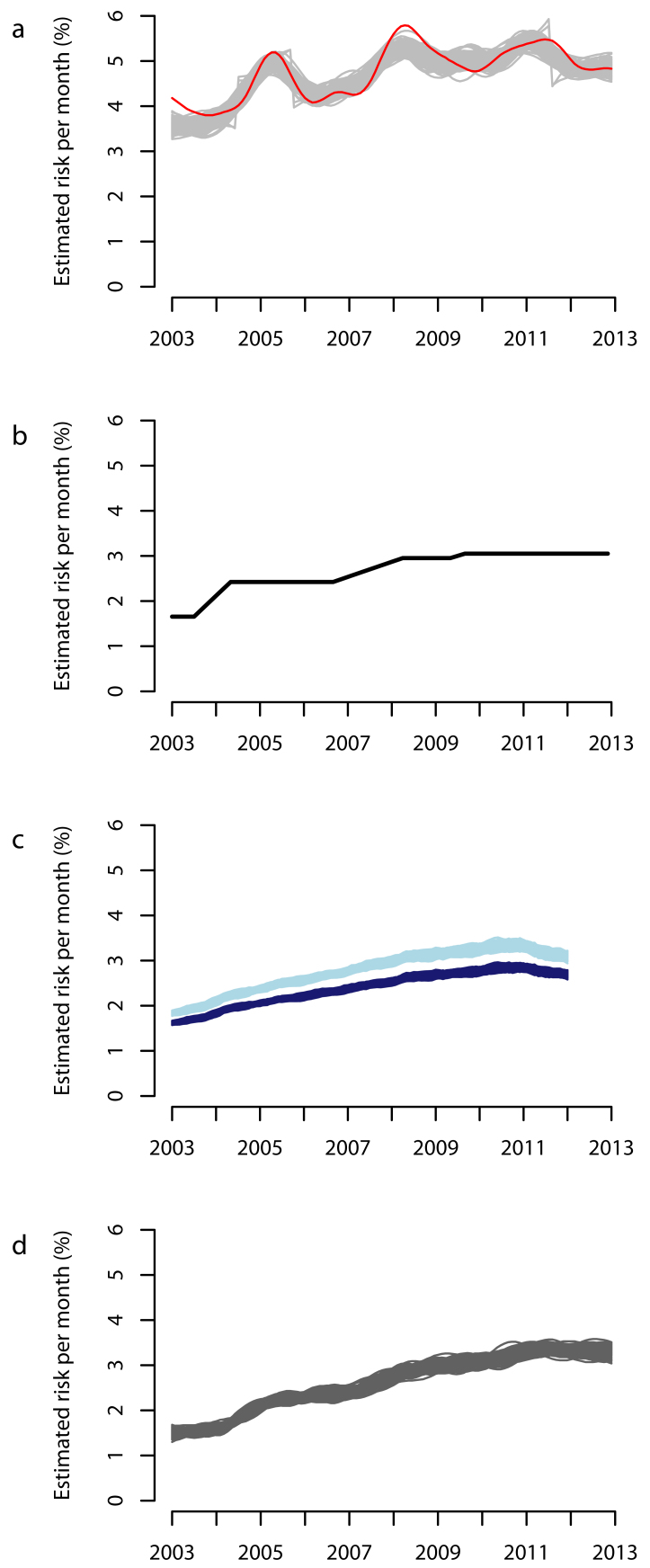
a) Current Defra method (smoothed % of OTF tested herds that are OTFW), red line corresponds to observed data and the grey lines correspond to the method applied to 100 simulations from the fitted model of herd testing and incidence. b) The underlying risk from the best fit model (x12). c) The adapted Cox method (x12) applied to the 100 model simulations either assuming no heterogeneity in risk (dark blue lines) within testing frequency groups or that only 1/5 of herds in each testing frequency group were at risk of developing an incident infection (light blue lines). d) The new simple incidence-based method applied to 100 simulations that accounts for the time between routine herd tests (adjusted smoothed % of OTF tested herds that are OTFW).

Applying this current Defra method to 100 stochastic simulations from the best-fitting mathematical model of herd testing and infection incidence (Fig 1a grey lines) replicated the observed smoothed national incidence trend presented by Defra, except the model slightly underestimated the peak of the second fluctuation.

As found previously^7^, the true underlying incidence risk for each of one hundred simulations from the best fitting model was estimated to monotonically increase over time without fluctuations (Fig 1b). In the current fit, a small increase in risk was estimated to occur in the third quarter of 2009. Maximum likelihood estimates of parameters are given in Appendix Table A1 and a comparison of the updated underlying model-estimated incidence risk to our previous fit is shown in Appendix Fig A1.

The current Defra method of using the percentage of tests in OTF herds that result in OTFW breakdowns over time applied to the 100 stochastic simulations (Fig 1a, grey lines) did not correspond well with the estimated underlying risk, producing clear artefactual fluctuations. The incidence trend calculated from this method was also higher than the underlying risk by a factor of 1.8 (mean across time series).

The adapted Cox method^7^ applied to each of the 100 stochastic simulations provided an accurate representation of the underlying risk, whereby assuming heterogeneity in risk (assuming one fifth of herds in each testing frequency group were at risk of a herd breakdown) provided a closer representation (light blue lines) than assuming no heterogeneity in risk (dark blue lines) (Fig 1c).

The incidence trends inferred by the smoothed new simple incidence-based method applied to each of the 100 stochastic simulations (Fig 1d) also reflected the estimated underlying risk without pronounced fluctuations and no requirement of an assumption regarding heterogeneity of risk, although the timings of the changes in risk were more lagged than with the adapted Cox method.

## Discussion

There is an urgent need to communicate clearly the incidence trends of bovine TB in GB. A large debate continues as to whether additional control methods (such as badger culling^5^ or vaccination^6^ ) are required to reduce the national incidence of cattle TB. The national reporting system currently uses a method which is liable to artefactual fluctuations. These can lead the public to believe that the incidence is declining when in fact it could instead be an artefact of the methods used.^7^ i.e. for a given underlying incidence risk which does not contain fluctuations but monotonically increases (as estimated in this work) the national reporting system can produce artefactual fluctuations which do not represent the underlying trend. We have previously shown that an adapted method proposed by Cox^7^^,^ 8 provides an accurate representation of the estimated risk, but this method is mathematically complex and it may not be sufficiently straightforward for use in the national statistics. We therefore developed a new simple incidence-based method to represent the underlying incidence trend from the national targeted surveillance data.

Here we have shown that new proposed method that adjusts the current reporting method for the time period between routine herd tests provides a better representation of the estimated underlying trends compared to the current Defra method. This method weights the test-positive tests with OTF status withdrawn by the time period since the last TB herd test, such that herds on four-yearly testing contribute a quarter compared to annually tested herds to the incidence at a given time.

The incidence trends obtained from both this new incidence-based method and the adapted Cox method are similar in magnitude to the underlying risk, whereas the incidence of bovine TB obtained from the current Defra method is generally higher (by a factor of 1.8) due to the overrepresentation of high risk herds (an inevitable feature of a targeted surveillance program). The expansion of the herds annually tested over time has caused the extent of the bias toward high risk herds to vary over time. The new incidence-based method does not provide as accurate a representation of the underlying risk trends as the adapted Cox method, because the timings of the change in risk are delayed, but importantly the method does not result in pronounced artefactual oscillations. Therefore, this method would be advantageous compared to the current reporting method.

## Competing Interests

The authors have declared that no competing interests exist.

## Figures and Tables

**Figure:**
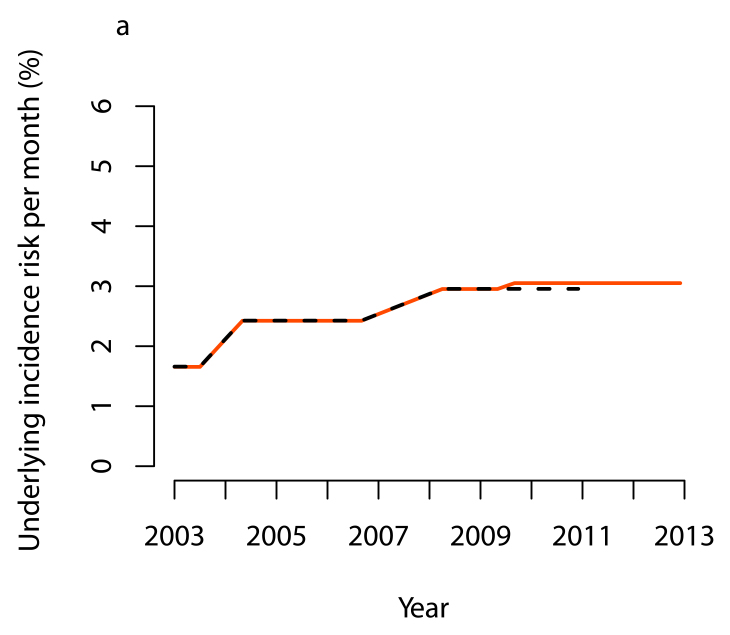
Comparison of a) the underlying incidence risk from the best fit model from 2003-2010 (black dashed line) compared to b) the underlying incidence risk from the updated best fit model 2003-2012 (red line).

## Appendix 1

### Appendix Table A1

**Estimated parameters of the best fit model of cattle herd incidence and testing frequencies fitted to data from Great Britain on the monthly number of herds tested and number of these with officially TB free status withdrawn in 2003-2012.** Full details of the model structure and parameter definitions are described previously.^7^

Model Parameter	Estimate
Proportion of herds with a relatively high risk of developing an incident infection at the start of 2003	0.16
Probability of a herd entering a higher risk category at each time step when time ≥ *z_1_* and <* z_2_*	0.015
Probability of a herd entering a higher risk category at each time step when time is ≥ *z_3_* and < *z_4_*	0.005
Probability of a herd entering a higher risk category at each time step when time is ≥ *z_5_* and < *z_6_*	0.004
*z_1_* Time threshold at which incidence risk increases	September 2003
*z_2 _*Time at which incidence risk stops increasing	July 2004
*z_3_* Time at which incidence risk starts increasing again	November 2006
*z_4_* Time at which incidence risk stops increasing	June 2008
*z_5_* Time at which incidence risk starts increasing again**	July 2009
*z_6_* Time at which incidence risk stops increasing**	November 2009
Threshold number of cumulative incident infections within a parish which when reached, the frequency interval of parish herd testing is changed to two-yearly testing	1 incident infection
Threshold number of cumulative incident infections within a parish which when reached, the frequency interval of parish herd testing is changed to annual testing	1 incident infection
Proportion of high-risk herds at the start of 2003 who are on annual testing	0.18
The first time after the beginning of 2003 when testing frequencies are changed	November 2004
The period between changing of testing frequencies	2 years and 11 months

## Appendix 2

### Published statistics on the incidence of tuberculosis (TB) in cattle in Great Britain

(Downloaded from https://www.gov.uk/government/publications/incidence-of-tuberculosis-tb-in-cattle-in-great-britain on 01/09/2013)

**Table:**

Updated on:	14/08/2013		Contact:	tbstatistics@defra.gsi.gov.uk				
Next update:	11/09/2013		Media Enquiries to:	0207 238 6007 (Press Office)				
			Source:	Animal Health and Veterinary Laboratories Agency (AHVLA) work management IT support system (SAM)				
**TB in Cattle in Great Britain**								
			Classification:	Public Domain		Units:	Various	
								
**TABLE 1: TB INCIDENTS IN GREAT BRITAIN - HERDS**								
			Total tests on herds	Herds not officially TB free **(non-OTF herds)**	Tests on officially TB free herds** (OTF)**	Of which: New herd incidents	Of which: officially TB free withdrawn **(OTFW)**	% of tests on officially TB free herds which resulted in officially TB free status being withdrawn
								
			(1)	(2)	(3)	(4)	(5)	(6)
	1996		36,314	1,589	34,812	1,075	490	1.4%
	1997		34,065	1,632	32,295	1,195	540	1.7%
	1998		37,046	2,077	34,502	1,514	787	2.3%
	1999		41,365	2,374	38,338	1,661	967	2.5%
	2000		40,669	2,482	37,184	1,738	1,135	3.1%
	2001	*	13,187	1,697	11,118	802	571	5.2%
	2002	**	49,709	4,167	43,641	3,323	2,042	4.7%
	2003		56,208	5,460	47,568	3,214	1,789	3.8%
	2004		56,836	5,220	49,027	3,341	1,934	4.0%
	2005		55,887	5,669	46,725	3,665	2,308	4.9%
	2006		64,457	5,859	56,051	3,530	2,303	4.1%
	2007		64,145	6,582	54,856	4,188	2,546	4.7%
	2008		66,432	7,935	54,854	5,011	3,093	5.6%
	2009	(prov)	72,205	8,386	58,894	4,599	2,847	4.9%
	2010	(prov)	74,474	7,964	61,587	4,723	3,013	4.9%
	2011	(prov)	76,662	8,252	62,493	4,909	3,109	5.2%
	2012	(prov)	88,566	9,067	73,656	5,192	3,465	4.8%
	2013	(prov)	42,441	7,533	35,337	2,246	1,404	4.0%
								
1996	Jan		4,152	638	4,038	120	57	1.4%
	Feb		4,968	660	4,843	108	54	1.1%
	Mar		5,082	734	4,951	134	54	1.2%
	Apr		3,588	780	3,444	114	51	1.5%
	May		3,327	789	3,155	90	30	1.0%
	Jun		2,022	762	1,881	74	36	1.9%
	Jul		1,665	729	1,515	82	30	2.0%
	Aug		1,724	668	1,600	52	22	1.4%
	Sep		1,639	653	1,522	63	33	2.2%
	Oct		2,256	574	2,148	70	30	1.4%
	Nov		3,181	499	3,086	91	50	1.6%
	Dec		2,710	502	2,629	77	43	1.7%
1997	Jan		3,864	543	3,739	106	51	1.4%
	Feb		4,046	565	3,929	95	48	1.2%
	Mar		3,794	597	3,679	96	34	0.9%
	Apr		3,507	697	3,343	159	69	2.1%
	May		3,020	701	2,838	96	36	1.3%
	Jun		2,182	729	1,998	107	30	1.5%
	Jul		1,866	690	1,710	78	26	1.6%
	Aug		1,590	663	1,426	63	31	2.2%
	Sep		1,756	661	1,601	106	49	3.1%
	Oct		2,541	653	2,377	86	47	2.0%
	Nov		2,994	658	2,863	112	60	2.1%
	Dec		2,905	651	2,792	91	59	2.1%
1998	Jan		4,133	707	3,934	144	82	2.1%
	Feb		4,363	770	4,180	145	76	1.8%
	Mar		4,137	861	3,956	176	89	2.3%
	Apr		3,692	905	3,445	138	63	1.9%
	May		3,011	894	2,779	122	53	1.9%
	Jun		2,323	931	2,094	141	61	3.0%
	Jul		2,298	912	2,032	107	57	2.9%
	Aug		1,664	859	1,467	100	64	4.4%
	Sep		1,708	835	1,508	96	51	3.4%
	Oct		2,899	813	2,672	112	54	2.0%
	Nov		3,521	841	3,298	132	83	2.5%
	Dec		3,297	812	3,137	101	54	1.7%
1999	Jan		4,447	852	4,223	139	77	1.8%
	Feb		4,794	924	4,569	186	111	2.5%
	Mar		4,750	1,052	4,540	220	119	2.6%
	Apr		4,884	1,079	4,540	165	89	2.0%
	May		3,322	1,093	3,064	147	83	2.7%
	Jun		2,318	1,120	2,053	136	71	3.5%
	Jul		2,834	1,055	2,467	108	59	2.4%
	Aug		1,879	975	1,657	86	52	3.1%
	Sep		2,153	918	1,904	87	58	3.1%
	Oct		2,713	849	2,452	97	59	2.4%
	Nov		3,609	856	3,414	177	113	3.3%
	Dec		3,662	839	3,455	113	76	2.2%
2000	Jan		4,304	943	4,037	199	128	3.2%
	Feb		4,915	1,017	4,652	200	129	2.8%
	Mar		5,656	1,091	5,333	175	110	2.1%
	Apr		3,818	1,116	3,497	141	86	2.5%
	May		3,515	1,114	3,198	138	93	2.9%
	Jun		2,876	1,087	2,531	121	65	2.6%
	Jul		2,193	1,023	1,874	104	69	3.7%
	Aug		2,104	987	1,828	110	64	3.5%
	Sep		2,219	950	1,920	113	77	4.0%
	Oct		2,247	935	2,029	132	92	4.6%
	Nov		3,657	979	3,340	159	112	3.4%
	Dec		3,165	991	2,945	146	110	3.8%
2001	Jan		3,791	1,102	3,496	207	142	4.1%
	Feb	*	4,340	1,104	3,997	144	91	2.4%
	Mar	*	601	1,007	538	15	13	2.4%
	Apr	*	386	1,008	302	35	27	8.9%
	May	*	360	1,003	232	28	16	6.9%
	Jun	*	332	974	206	32	22	10.7%
	Jul	*	358	950	193	35	19	10.1%
	Aug	*	355	906	187	19	9	5.1%
	Sep	*	364	873	194	42	30	15.5%
	Oct	*	398	877	257	65	53	20.6%
	Nov	*	841	879	638	76	60	9.5%
	Dec	**	1,061	916	878	104	89	10.1%
2002	Jan	**	2,765	1,045	2,515	202	136	5.4%
	Feb	**	4,437	1,324	4,185	375	248	5.9%
	Mar	**	5,535	1,589	5,253	348	245	4.7%
	Apr	**	5,164	1,918	4,649	397	250	5.4%
	May	**	5,509	2,144	4,916	320	171	3.5%
	Jun	**	2,685	2,190	2,238	194	95	4.3%
	Jul	**	3,030	2,304	2,441	235	129	5.3%
	Aug	**	2,918	2,298	2,261	182	111	5.0%
	Sep	**	2,752	2,298	2,139	206	121	5.7%
	Oct	**	3,787	2,339	3,141	249	144	4.7%
	Nov	**	6,217	2,406	5,532	329	201	3.6%
	Dec		4,910	2,452	4,371	286	191	4.4%
2003	Jan		7,032	2,620	6,227	374	219	3.5%
	Feb		6,181	2,678	5,449	337	191	3.5%
	Mar		6,213	2,839	5,499	430	238	4.4%
	Apr		4,826	2,912	4,066	333	174	4.3%
	May		5,332	2,833	4,414	238	130	3.0%
	Jun		3,844	2,768	2,999	260	132	4.4%
	Jul		3,720	2,612	2,989	206	96	3.2%
	Aug		2,991	2,372	2,327	118	58	2.5%
	Sep		3,390	2,300	2,713	216	127	4.7%
	Oct		4,106	2,188	3,401	201	113	3.4%
	Nov		4,499	2,158	3,890	264	171	4.4%
	Dec		4,074	2,145	3,594	237	140	3.9%
2004	Jan		6,385	2,216	5,692	337	200	3.5%
	Feb		5,973	2,284	5,317	312	183	3.4%
	Mar		5,994	2,488	5,416	439	239	4.4%
	Apr		6,350	2,564	5,533	344	176	3.2%
	May		4,560	2,584	3,877	265	139	3.6%
	Jun		3,555	2,583	2,931	272	134	4.6%
	Jul		3,949	2,457	3,106	170	95	3.1%
	Aug		2,984	2,369	2,394	196	117	4.9%
	Sep		3,132	2,226	2,490	159	96	3.9%
	Oct		3,832	2,147	3,245	216	135	4.2%
	Nov		5,212	2,245	4,641	369	246	5.3%
	Dec		4,910	2,223	4,385	262	174	4.0%
2005	Jan		5,793	2,431	5,166	429	270	5.2%
	Feb		6,308	2,698	5,561	461	290	5.2%
	Mar		5,919	2,929	5,281	423	293	5.6%
	Apr		6,357	3,050	5,238	384	227	4.3%
	May		4,044	2,983	3,202	257	163	5.1%
	Jun		3,344	2,875	2,443	218	134	5.5%
	Jul		3,346	2,731	2,504	174	100	4.0%
	Aug		2,772	2,602	2,078	194	117	5.6%
	Sep		3,591	2,514	2,819	226	132	4.7%
	Oct		3,649	2,493	3,059	276	174	5.7%
	Nov		5,058	2,573	4,348	340	212	4.9%
	Dec		5,706	2,584	5,026	283	196	3.9%
2006	Jan		6,405	2,714	5,638	386	246	4.4%
	Feb		6,859	2,721	6,071	315	219	3.6%
	Mar		7,821	2,813	7,001	366	229	3.3%
	Apr		6,014	2,734	5,207	265	171	3.3%
	May		5,146	2,725	4,426	282	183	4.2%
	Jun		4,466	2,636	3,713	233	150	4.1%
	Jul		3,705	2,549	3,073	222	142	4.6%
	Aug		3,277	2,436	2,705	183	109	4.1%
	Sep		4,434	2,426	3,709	285	196	5.3%
	Oct		4,421	2,462	3,899	345	234	6.0%
	Nov		6,178	2,557	5,493	357	236	4.3%
	Dec		5,731	2,597	5,116	291	188	3.7%
2007	Jan		6,453	2,850	5,693	458	268	4.7%
	Feb		6,982	2,956	6,160	384	211	3.4%
	Mar		8,350	3,120	7,421	437	250	3.4%
	Apr		6,352	3,246	5,474	416	244	4.5%
	May		5,348	3,212	4,514	298	178	4.0%
	Jun		4,560	3,066	3,680	278	148	4.0%
	Jul		3,826	3,029	3,042	285	155	5.1%
	Aug		2,905	2,863	2,297	175	96	4.2%
	Sep		3,601	2,851	2,935	270	173	5.9%
	Oct		4,298	3,039	3,690	447	303	8.2%
	Nov		6,524	3,106	5,662	402	282	5.0%
	Dec		4,946	3,165	4,288	338	238	5.6%
2008	Jan		6,593	3,398	5,719	474	313	5.5%
	Feb		7,398	3,504	6,353	473	322	5.1%
	Mar		5,872	3,602	5,137	427	300	5.8%
	Apr		6,909	3,925	5,697	594	366	6.4%
	May		5,908	3,901	4,901	390	240	4.9%
	Jun		4,319	3,946	3,309	375	194	5.9%
	Jul		4,236	3,973	3,171	359	174	5.5%
	Aug		3,768	3,947	2,934	305	178	6.1%
	Sep		4,105	4,019	3,206	381	220	6.9%
	Oct		5,603	4,047	4,582	426	283	6.2%
	Nov		6,193	4,116	5,155	448	275	5.3%
	Dec	(prov)	5,528	4,136	4,690	359	228	4.9%
2009	Jan	(prov)	7,894	4,320	6,552	533	301	4.6%
	Feb	(prov)	7,181	4,339	6,058	438	252	4.2%
	Mar	(prov)	7,551	4,542	6,423	572	330	5.2%
	Apr	(prov)	7,523	4,563	6,127	440	256	4.2%
	May	(prov)	6,019	4,464	4,822	359	207	4.3%
	Jun	(prov)	4,609	4,342	3,538	366	212	6.0%
	Jul	(prov)	4,889	4,116	3,564	294	193	5.4%
	Aug	(prov)	3,953	3,791	3,048	214	139	4.6%
	Sep	(prov)	4,413	3,654	3,535	306	209	6.0%
	Oct	(prov)	6,270	3,522	5,191	333	235	4.5%
	Nov	(prov)	6,139	3,637	5,135	454	308	6.0%
	Dec	(prov)	5,764	3,570	4,901	290	205	4.2%
2010	Jan	(prov)	6,852	3,675	5,758	434	284	5.0%
	Feb	(prov)	8,114	3,766	6,916	440	277	4.0%
	Mar	(prov)	8,121	3,905	7,144	527	328	4.6%
	Apr	(prov)	8,834	3,933	7,365	428	259	3.5%
	May	(prov)	5,833	3,879	4,764	388	218	4.6%
	Jun	(prov)	4,059	3,875	3,104	391	209	6.8%
	Jul	(prov)	5,086	3,683	3,733	247	161	4.4%
	Aug	(prov)	3,956	3,517	3,032	288	181	6.0%
	Sep	(prov)	4,939	3,451	3,937	359	249	6.4%
	Oct	(prov)	6,243	3,475	5,231	404	277	5.3%
	Nov	(prov)	6,664	3,640	5,687	525	377	6.7%
	Dec	(prov)	5,773	3,615	4,916	292	193	3.9%
2011	Jan	(prov)	7,830	3,881	6,531	538	350	5.4%
	Feb	(prov)	7,910	4,025	6,653	464	302	4.6%
	Mar	(prov)	8,615	4,164	7,486	510	312	4.2%
	Apr	(prov)	7,035	4,143	5,694	397	241	4.3%
	May	(prov)	6,142	4,199	4,898	467	*270 - 285*	*5.5% - 5.8%*
	Jun	(prov)	4,675	4,121	3,415	347	*202 - 212*	*5.9% - 6.2%*
	Jul	(prov)	4,656	4,009	3,388	300	*183 - 225*	*5.4% - 6.6%*
	Aug	(prov)	4,231	3,864	3,209	292	*188 - 248*	*5.9% - 7.7%*
	Sep	(prov)	5,900	3,688	4,660	251	*166 - 183*	*3.6% - 3.9%*
	Oct	(prov)	6,010	3,786	5,026	421	*281 - 298*	*5.6% - 5.9%*
	Nov	(prov)	6,563	4,013	5,570	489	*331 - 356*	*5.9% - 6.4%*
	Dec	(prov)	7,095	4,137	5,963	433	*283 - 306*	*4.7% - 5.1%*
2012	Jan	(prov)	8,195	4,360	6,996	485	*326 - 334*	*4.7% - 4.8%*
	Feb	(prov)	9,062	4,498	7,702	465	*315 - 326*	*4.1% - 4.2%*
	Mar	(prov)	11,717	4,760	10,282	591	*398 - 406*	*3.9% - 3.9%*
	Apr	(prov)	7,891	4,882	6,536	408	*249 - 254*	*3.8% - 3.9%*
	May	(prov)	7,306	4,979	5,994	448	*259 - 266*	*4.3% - 4.4%*
	Jun	(prov)	5,426	4,941	4,177	326	*199 - 202*	*4.8% - 4.8%*
	Jul	(prov)	5,061	4,922	3,860	315	*216 - 218*	*5.6% - 5.6%*
	Aug	(prov)	5,422	4,973	4,244	357	*245 - 250*	*5.8% - 5.9%*
	Sep	(prov)	5,881	5,028	4,742	376	*264 - 272*	*5.6% - 5.7%*
	Oct	(prov)	6,816	5,214	5,748	452	*328 - 333*	*5.7% - 5.8%*
	Nov	(prov)	9,060	5,450	7,680	570	*393 - 401*	*5.1% - 5.2%*
	Dec	(prov)	6,729	5,545	5,695	399	*273 - 275*	*4.8% - 4.8%*
2013	Jan	(prov)	8,750	5,795	7,339	508	*307 - 319*	*4.2% - 4.3%*
	Feb	(prov)	9,004	5,907	7,561	409	*278 - 283*	*3.7% - 3.7%*
	Mar	(prov)	9,257	6,029	7,990	496	*306 - 314*	*3.8% - 3.9%*
	Apr	(prov)	8,076	6,103	6,545	429	*275 - 282*	*4.2% - 4.3%*
	May	(prov)	7,354	6,039	5,902	404	*238 - 244*	*4.0% - 4.1%*
								
								
**Notes:-** The data are a snapshot extracted from Sam. Data for 2009 onwards will remain provisional and subject to revision until all culture results are available and final data validation has been carried out. The herd incidence rates for the latest months are given as a range because a number of incidents are still unclassified, so data for these months should be treated as provisional results. TB incidents remain unclassified if at the end of the period covered by this notice they had not been designated OTFW, but were still ongoing and the herd could have its OTF status withdrawn if further testing revealed one or more animals with post-mortem evidence of TB.								
								
(1)	Herds for which tuberculin skin testing is carried out on at least one animal during the period shown.							
(2)	Herds that had lost their OTF status at some time during the period shown due to a TB incident.							
(3)	Any test carried out in an OTF herd during the period shown.							
(4)	Herds which were previously OTF but either had cattle that reacted to a tuberculin test or had a tuberculous animal disclosed by routine meat inspection at slaughter, during the period shown.							

(5)	New herd incidents (column 4) where OTF status was withdrawn from the herd.							
(6)	Column 5 as a percentage of column 3.							
*	Data for 2001 are not comparable with other years. During the outbreak of Foot and Mouth Disease, TB testing was significantly reduced and necessarily targeted to areas of higher risk.							

**	Data for 2002 are not comparable with other years. Testing resources were concentrated on herds which were overdue their tests (because of the backlog caused by the Foot and Mouth Disease outbreak).							

## References

[1] Krebs JR et al. Bovine tuberculosis in cattle and badgers. (HMSO, London) 1997

[2] Defra. Monthly publication of national statistics on the incidence of tuberculosis (TB) in cattle to end June 2013 for Great Britain (Accessed September 2013)

[3] Defra. Request for information: various bovine TB costs (2008-2013) (Accessed September 2013)

[4] Defra. Changes to bovine TB surveillance: bovine TB information note 04/12 (Accessed September 2013)

[5] Defra. Bovine tuberculosis: The government's approach to tackling the disease and consultation on a badger control policy (Accessed September 2013)

[6] Welsh Government. Badger vaccination underway (Accessed September 2013)

[7] Blake IM, Donnelly CA. Estimating risk over time using data from targeted surveillance systems: application to bovine tuberculosis in Great Britain. Epidemics. 2012 Dec;4(4):179-86. PubMed PMID:23351370. 23351370 10.1016/j.epidem.2012.09.003

[8] Cox DR. An Industrial monitoring problem. Quality Engineering. 2010 22:73-77

